# Biological parameters determining the effectiveness of monitoring of HIV / AIDS infected patients in Morocco

**DOI:** 10.4314/ahs.v23i2.12

**Published:** 2023-06

**Authors:** Mohamed Echchakery, Ali Boumezzough, Samia Boussaa

**Affiliations:** 1 Hassan First University of Settat, Higher Institute of Health Sciences, Laboratory of Sciences and Health Technologies, Epidemiology and biomedical unit, 26000 Settat. University Complex, Casablanca Road km 3.5 B. P 555 Settat, Morocco; 2 Laboratory of Medical Analysis, Ibn Zohr Regional Hospital Center, Marrakech, Morocco; 3 Microbial Biotechnologies, Agrosciences and Environment Laboratory (BioMAgE), Faculty of Sciences Semlalia, Cadi Ayyad University, Marrakech, Morocco; 4 ISPITS-Higher Institute of Nursing Professions and Health Techniques, Ministry of Health and social protection, 10000 Rabat, Morocco

**Keywords:** HIV, viral load, CD4, therapeutic failure, antiretroviral treatment, Morocco

## Abstract

**Background & objective:**

Human Immunodeficiency Virus (HIV) remains one of the world's most serious health challenges. The development of therapeutic regimens has significantly increased survival and reduced HIV-associated morbidities in HIV-infected individuals. However, some people living with HIV may not respond as expected, resulting in treatment failure. The objective of this study is to identify and characterize, by immunological (T-cell CD4) and virological (viral load) parameters, HIV infected patients with therapeutic failure in Morocco.

**Methods:**

Prospective cross-sectional studies were conducted over a 5-years period (between January 2015 and December 2019) at the referral center of Ibn Zohr Hospital, Marrakech, Morocco. A total, of 1088 HIV-infected patients diagnosed by the rapid test (Immunochromatography) in addition to Western Blot analysis, was recruited. All patients were under the antiretroviral therapy (ART) for at least six months and followed every six months. Sociodemographic, clinical, and biological data as well as information on patient adherence were collected.

**Results:**

Out of 1088 patients, 92.46% were under treatment based on non-nucleoside reverse transcriptase inhibitors (NNRTIs) including 26.20% first line first intention and 66.26% first line second intention, and 7.54% of patients on a protease inhibitor (PI) therapy. Regarding the immunological and virological status, 76% of HIV-infected patients had a CD4 count > 200 cells/µl and 24% had a CD4 count < 200 cells / µl, while 69.5% had an undetectable viral load and 30.05% had a detectable viral load (including 11.86% with viral load < 1000 copies / ml and 18.20% viral load > 1000 copies / ml) (P-values < 0.05).

**Conclusion:**

In our study, we showed a therapeutic failure rate of 18.2% in HIV-infected patients under treatment in Marrakech region. These failures were mainly related to poor adherence and low CD4+ rates at the initiation of treatment. We concluded that immunological monitoring alone is insufficient to predict virological suppression and therapeutic success. Consequently, we recommend the HIV plasma viral load test be accessible as a routine exam.

## Introduction

Human Immunodeficiency Virus (HIV) remains one of the greatest global health challenges of our time; it is estimated that 36.7 million people are currently living with HIV globally, with at least 1.8 million more becoming newly infected each year 1.

Acquired immunodeficiency syndrome (AIDS) is a chronic infection caused by the Human Immunodeficiency Virus (HIV). The world is making tremendous progress towards curbing the course of the global HIV/ AIDS pandemic. Since 2005, AIDS-related deaths have fallen globally by 48 percent; and since 2010, new HIV infections in children have fallen by 47 percent 2. However, while significant progress has been made in the global fight against HIV/AIDS, there is still much more to be done to develop holistic, sustainable, and measurable solutions that deliver long-term impact 2.

The HIV retrovirus exists in two forms (HIV-1 and HIV-2). HIV type 1 (HIV-1) is the most contagious and widely distributed in the world. This virus attacks the body's immune system, specifically the CD4 (Cluster of differentiation 4) cells (T cells). The absence of antiretroviral therapy in HIV-positive patients leads to an increase in the number of viral RNA copies in the blood accompanied by a decrease in CD_4_ count, which gives the infection the chance to progress to the AIDS stage and therefore the appearance of opportunistic diseases, lymphoproliferative neoplasia and neurological disorders 3.

HIV continues to be a major global public health issue, having claimed more than 32 million lives so far 4;5. In 2018, 770 000 people died from HIV-related causes globally 4;5. There were approximately 37.9 million people living with HIV at the end of 2018 with 1.7 million people becoming newly infected in 2018 globally 4;5. 62% of adults and 52% of children living with HIV were receiving lifelong antiretroviral therapy (ART) in 2018 4;5. The World Health Organization (WHO) African Region is the most affected region, with 25.7 million people living with HIV in 2018 4;5. The African region also accounts for almost two thirds of the global total of new HIV infections. It is estimated that currently 79% of people with HIV know their status 4;5. In 2018, 23.3 million people living with HIV were receiving ART globally 4; 5.

In Morocco, in 2018, the epidemiological estimate was 21,000 people living with HIV (adults and children), 900 new HIV infections per year and 350 AIDS-related deaths. In 2019, the number of new cases of HIV infection notified was 1,385 throughout the country, bringing the number of cumulative cases to 17,000 since the start of the epidemic in 1986. A total of 41% have been notified for the past five years. Nearly two-thirds (63%) of HIV/AIDS infection cases in the whole country were reported in three regions: 23% in the Souss-Massa region, 20% in the Marrakech-Safi region, and 20% in the Casablanca-Settat region 6.

There are currently different classes of anti-human immunodeficiency virus drugs, which act at different stages of HIV replication including entry and fusion inhibitors, HIV reverse transcriptase inhibitors, inhibitors of integrase and protease inhibitors. Up to 30% of Highly Active Antiretroviral Therapy (HAART)-infected patients do not show a marked increase in CD_4_^+^ T-cell counts. There is still concern that immune recovery may not be complete once CD_4_^+^T cells have decreased below 200 cells/µl 7.

Situations of virologic failure should be detected by regular viral load checks (M1, M3, M6 then every 6 months), so that causes are identified and corrected early to restore virologic success and prevent the accumulation of resistance mutations. The emergence of resistance mutations on antiretroviral therapy has greatly diminished over the past 15 years due to the power of current antiretrovirals (ARVs) and regular virological monitoring 8. Multidrug-resistant viruses are currently mainly found in HIV-positive patients with complex and old therapeutic histories. In most of these cases, a suitable treatment makes it possible to obtain an undetectable viral load.

In Morocco, virologic and immunologic failure was noted among HIV infected patients. The objective of this study is to determine the prevalence of therapeutic failure among patients living with HIV in Morocco and its immunological (T-cell CD^4^) and virological (viral load) characterization.

## Materials and methods

### Design and study participants

An exploratory and retrospective descriptive study was carried out in the regional hospital center (Ibn Zohr hospital), infectious disease service and medical analysis laboratory, Marrakech, Morocco. It is a referral center in the Marrakech-Safi region for the diagnosis, confirmation and monitoring of patients living with HIV.

Among patients followed and under treatment, the inclusion criteria for the study population recruitment were:

- Patients in therapeutic failure,

- Under ARV treatment for at least 6 months,

- Therapeutic failure: defined by a viral load >1000 copies / mL.

After purification by the remaining inclusion criteria, 1088 records of patients on HAART, were recruited for immunological and virological analysis and were followed between January 2015 and December 2019.

### Data collection

Clinical and biological information of patients infected with HIV / AIDS participating in this study were obtained from the patient files. These record the value of the viral load and the number of CD_4_^+^ lymphocytes each time it is performed, every six months according to the protocol. In addition, individual variations were noted; the appearance of opportunistic infections, adverse events associated with antiretroviral such as anemia, rashes, peripheral neuropathy, pancreatitis. The patient adherence to HAART, was considered optimal if the self-reported percentage of monthly antiretroviral intake is greater than 95%.

### Ethical considerations

This study is part of a project approved by the Hospital University Ethics Committee under approval number 020/2016, for carrying out an epidemiological study on patients living with HIV in the Marrakech-Safi region. In addition, the authorization was obtained from the Regional Health Department to examine clinical registries and recruit participants.

A consent form was explained and signed by all adult participants and parents or guardians for adolescent patients.

### Sample collection and testing

On the day of the control and recovery of the HAART treatments, the blood samples were taken on two EDTA tubes to assess CD4 count and HIV viral load. New patients referred by different departments were screened by rapid test and then confirmed by western blot.

### Rapid HIV Testing

Early diagnosis of acute HIV infection via rapid HIV testing can identify patients who will benefit from antiretroviral treatment, which has been shown to delay the progression to AIDS and death and reduce the transmission of HIV [9]. Rapid HIV testing may also be useful to quickly confirm the diagnosis of HIV infection in patients who present with an AIDS-defining illness but have unknown HIV status.

Diagnostic Kit for HIV (1+2) Antibody (Colloidal Gold) V2 is an in vitro, visually read test for the qualitative determination of antibodies against HIV-1 and HIV-2 in human serum, plasma, venous and capillary whole blood. This rapid test is intended for use as an aid to screening and to detect antibodies to HIV (1 + 2) in individuals suspected of being infected with HIV.

### Western Blot Test

A Western blot test is typically used to confirm a positive HIV diagnosis. During the test, a blood sample is used to detect HIV antibodies, not the HIV virus itself. The Western blot test separates the blood proteins and detects the specific proteins (called HIV antibodies) that indicate an HIV infection. The Western blot is used to confirm a positive ELISA, and positive rapid tests are 99.9% accurate.

### Plasma HIV-1 RNA (Viral Load)

In this study to determine the plasma HIV-1 Viral Load, we used the GeneXpert® Instrument Systems automated machine and integrate specimen preparation, nucleic acid extraction, and amplification, and detection of the target sequence in simple or complex specimens using real-time reverse transcriptase PCR (RT-PCR). The systems consist of an instrument, a personal computer with preloaded software for running tests and viewing the results.

GeneXpert HIV-1 Viral Load is an in vitro reverse transcriptase polymerase chain reaction (RT-PCR) assay for the detection and quantification of Human Immunodeficiency Virus type 1 (HIV-1) RNA in human plasma from HIV-1 infected individuals, using the automated GeneXpert Instrument Systems. The assay can quantify HIV-1 RNA over the range of 40 to 10,000,000 copies/mL. GeneXpert HIV-1 Viral Load is intended for use as an aid in assessing viral response to antiretroviral treatment as measured by changes in plasma HIV-1 RNA levels.

### Quality control

Each test includes a Sample Volume Adequacy (SVA) control, Internal Quantitative Standard High and Low (IQS-H and IQS-L), which is also a sample processing control, and a Probe Check Control (PCC).

## Results

**Figure 1:**
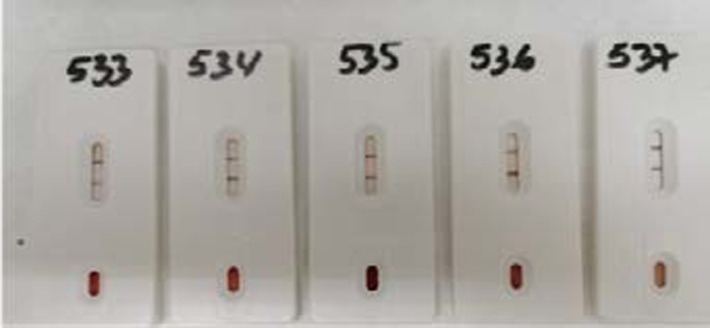
HIV positive test result by using rapid test cassette (probably presence of anti-HIV antibodies confirmation by western blot)

**Figure 2:**
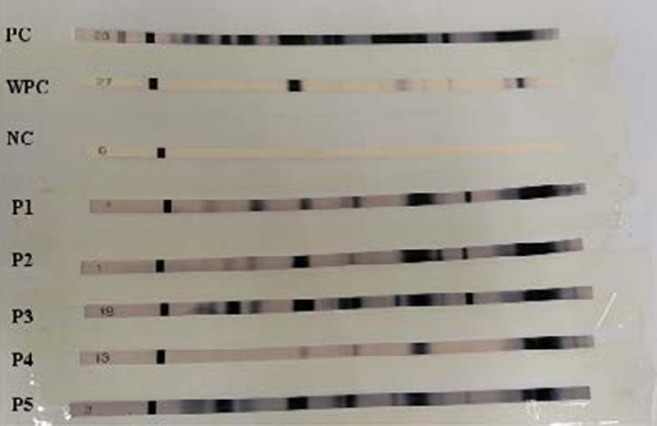
Sequential results from serum testing of a patient newly infected with HIV-1. PC: positive control; WPC: Weak Positive Control; NC: Negative Control; column P1-5: positive anti-HIV-1 serum; column 5: positive anti-HIV-1

A total of 1088 patients diagnosed HIV-positive by rapid test (Figure 1) and confirmed by Western Blot (Figure 2), were followed and included in the study. 53% (577) of the participants were males, 69.76% (750) were aged 25–45 years (Median age 32 (IQR: 28–40), 38.6% (420) were married, 75.37% (820) were heterosexual and 65.26% (710) lived outside Marrakech city (Safi, Al Haouz, Essaouira, Agadir, Casablanca, Laayoune) (Table 1).

**Table 1:** Sociodemographic characteristics of the study subjects

Sociodemographic characteristics	N	%
**Sex**		
Male	577	53
Female	511	47

**sex ratio (M/F)**	1,23	----------

**Age (years)**		
15–24	171	15,72
25–34	448	41,18
35–44	311	28,58
45–54	123	11,31
> 55	35	3,21

**Place of residence**		
Marrakech	378	34,74
Outside of Marrakech	710	65,26

**Marital Status**		
Never married	45	4,14
Married	420	38,60
Divorced	335	30,80
Widowed	288	26,46

**Sexual behaviour**		
Heterosexual	820	75,37
Homosexual	268	24,63

Out of 1088 HIV-infected patients, 92.46% (Table 2) were under treatment based on non-nucleoside reverse transcriptase inhibitors (NNRTIs) including 26.20% first line first intention and 66.26% first line second intention, and 7.54% of patients on a protease inhibitor (PI) therapy (according to the national recommendations of the first and second-line ARV drug regimens for adults and children 2013-2019).

**Table 2:** Basic information of first and second-line ART regimens for patients living with HIV of our subject of study

Regimen given at base line	NRTIs	NNRTIs	PI	N (1088)	n (%)
1st line 1st intention	TDF + 3TC	EFV	------------	281	26,20
				
	TDF + 3TC	NEV		04	

1st line 2nd intention	AZT + 3TC	EFV	------------	699	66,26
				
	AZT + 3TC	NEV		22	

2nd line 1st intention	TDF + 3TC	------	Aluvia	42	7,54
			
2nd line 2nd intention	AZT + 3TC	------		40	

Tenofovir (TDF), Lamivudine (3TC) and Efavirenz (EFV) or TDF / Zidovudine (AZT), 3TC and Nevirapine (NVP)s. Protease inhibitor: PI; Nucleoside / nucleotide reverse transcriptase inhibitors: NRTIs; Non-nucleoside reverse transcriptase inhibitors: NNRTIs ; Aluvia : Lopinavir (LPV) / Ritonavir

**Figure 3:**
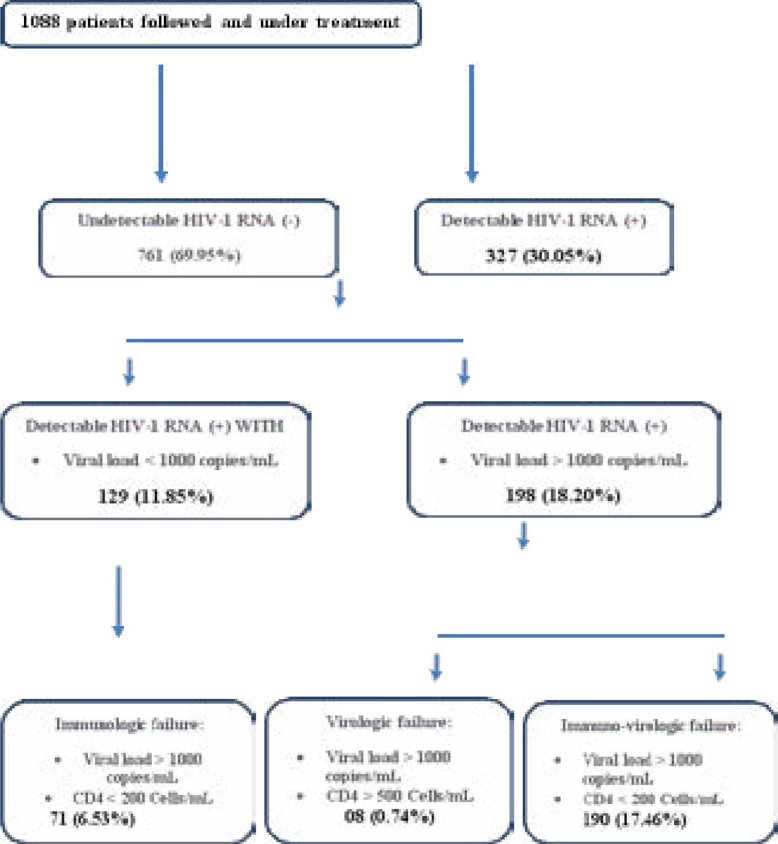
Workflow of the study

Regarding the immunological and virological status, 76% (827) of the 1088 HIV-infected patients had a CD4 count > 200 cells / µl, 24% (261) had a CD4 count <200 cells / µl and 69.5% (761) had an undetectable viral load and 30.5% 327 had a detectable viral load including 11.86% (129) viral load < 1000 copies / ml, 18.2% (198) viral load > 1000 copies / ml respectively (Table 3; Figure 3).

**Table 3:** Clinical, immunological and virological characteristics of the study subjects at enrolment to chronic HIV care, initiation of ART, and during follow-up in Ibn Zohr hospital Marrakech

	Male	Female	N	%
Patients Living with HIV	577	511	1088	100

Undetectable HIV-1 RNA (-)	409	352	761	69,5

Detectable HIV-1 RNA (+)	168	159	327	30,5

CD_4_^>^ 200 cells/µl	435	392	827	76

CD_4_< 200 cells/µl	142	119	261	24

**Figure 4:**
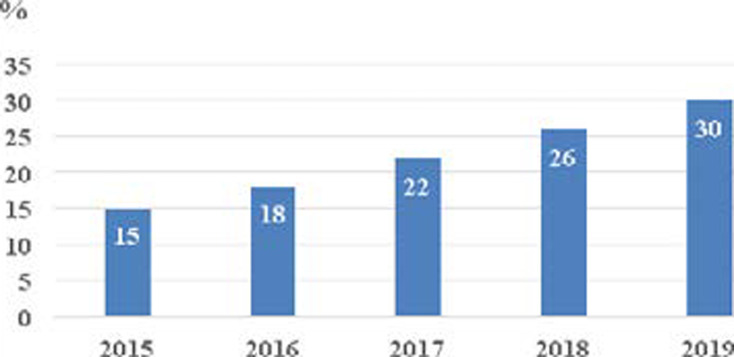
The distribution of failure in HIV-infected patients on ART during the period of study

Figure (4) presents the trends of the distribution of failure in HIV-infected patients on ART, between 2015 and 2019. The cumulative frequency percentage of patients with IF increases from 15% (on 2015) to 30% (on 2019). Among the cases where the clinical stage was recorded, 29.87% (325) were at clinical stage I / II and 70.13% (763) were at the clinical stage of AIDS (stage III / IV) according to the criteria of WHO (Table 4) 10.

**Table 4:** Virological and immunological failure according to the basic characteristics of patients infected with HIV / AIDS at the start of HAART

		Immunological failure		
		
Variable	Total	VF (n)	WVF (n)	*p-value*
**Gender**				
Male	577	24,61%	75,39%	**< 0,05**
Female	511	23,29%	76,71%	

**CD4 + count (cells / mL)**				
CD4 > 200 cells/µl	827	5,5%	76%	
CD4 <200 cells/µl	261	24%	0,96%	**< 0,05**

**Viral load (copies / mL)**				
> 1000 copies / mL	327	30%	0,10%	
<1000 copies / mL	761	0,95%	69,95	**< 0,05**

**Regimen given at base line**				
TDF 3TC EFV	281	74,17%	25,83%	
TDF 3TC NEV	04	99,63%	0,37%	
AZT 3TC EFV	699	35,75%	64,25%	
AZT 3TCNEV	22	97,98%	2,02%	
TDF 3TCL PV/ Ritonavir	42	96,14%	3,86%	
AZT 3TCL PV/ Ritonavir	40	96,32%	3,68%	

**Time on ART (months)**				
6–12	533	15,57%	84,43%	
13–24	717	18%	82%	**< 0,05**
25–36	877	22%	78%	
37–48	1060	25,67%	74,34%	
> 48	1088	30,05%	69,95%	

**Adherence**				
Fair/poor	82	2 (0,18%)	80 (7,35%)	**< 0,05**
Good	1006	26 (2,39%)	980 (90,07%)	

**WHO clinical stage at base line**				
I	27 (2.48)	04 (0.37)	23 (2.11)	
II	298 (27.39)	36 (3.31)	262 (24.08)	**< 0,05**
III	617 (56.71)	103 (9.47)	514 (47.24)	
IV	146 (13.42)	118 (10.84)	28 (2.57)	

The value of p corresponds to the comparison between WVF and FV (tests of χ^2^, Fisher's exact test or Mann-Whitney according to whether the variable is categorical or continuous). HAART: highly active anti-retroviral therapy. FV: virological failure. WVF: without virological failure. ARV: anti-retroviral. NNRTI: Non-nucleoside reverse-transcriptase inhibitor. IP: protease inhibitor. NRTI: Nucleoside reverse-transcriptase inhibitor. CD4: Cluster of differentiation 4

Monitoring viral load is the recommended approach in defining treatment failure, despite its costly nature. We compared the viral load of patients at 6 months and 12 months in each treatment regimen to assess therapeutic failure. The latter was calculated on the basis of two successive determinations of viral load with results > 1000 copies/ml, according to the WHO criteria. Data obtained at 6 months were used as a reference and those at 12 months was considered as the first point of time to assess the rate of virological failure (Table 4).

## Discussion

The first case of AIDS was recorded in Morocco in 1986. In 1988, the national AIDS Control Program was organized to provide a structure for responding to the disease 11. The mechanisms adopted were aimed at understanding and monitoring the spread of the disease, informing and protecting the population and providing treatment for people living with HIV. In November 1997, the first consensus conference was organized to develop national interecommendations for the diagnosis and care of people living with HIV. This consensus led to a first ministerial circular for the management of HIV infection in Morocco with the development of knowledge in this area. These recommendations have been updated several times.

In December 2013 and 2015, national recommendations were aligned with the “Consolidated guidelines for the use of Antiretrovirals (ARVs) for the prevention and treatment of HIV Infection” published by the World Health Organization Health (WHO) 12. HAART guideline in Morocco is given a combination of two Nucleoside reverse-transcriptase inhibitors (NRTI) and one Non-nucleoside reverse-transcriptase inhibitor (NNRTI). The first line regimens are Lamivudine (3TC) combined with Stavudine (d4T), zidovudine (AZT) or Tenofovir (TDF), and either nevirapine (NVP) or efavirenz (EFV) (Table 2). However, for anemic patients TDF substitutes AZT and for tuberculosis patients treated with Rifampicin EFV replaces NVP.

The aim of this study was to describe the virological (viral load) and immunological (T-cell CD_4_) failure in patients infected with HIV / AIDS and under ARV treatment in a total of 1088 patients. In this study, the magnitude of immunological and virological failure was found to be 24.73%, with only immunological, only virological, and both immunological and virological failures constituting 6.53%, 0.74%, and 17.46%, respectively. This magnitude is almost equivalent to the magnitude of failures reported in two Ethiopian studies, which is 21% and 22.7%^13^, ^14^ and a Tanzanian study: 25% 15. It is lower than the magnitude reported in India (26.1%), Malawi (48%) 16, 17, Thailand 33.5% 18, Nepal 35% 19 and Kenya 64.4% 20. In contrast, the found magnitude is higher than that reported in the study conducted in northern Ethiopia 6.5% 21, South Africa (Lesotho) where the magnitude was 11.8% 22 and also in the south of Ethiopia 11.5% 23, Jimma 9.8% 24, Addis Ababa 15.7% 25 and Gonder 15.1 % 26.

In our study, immunological failure was defined based on the WHO guideline 27, as drop of CD4+ count below 200 cells/µL following clinical failure, or persistent CD4+ count below 100 cells/µL. This research identifies that patient with baseline CD4 counts < 200 cells / mm^3^ are more likely to have virologic and immunologic, as well as clinical, failure (p- value < 0,05) than patients whose CD4 count is greater than> 200 cells / mm3. However, several researches aforementioned 15,20,23,24,26,28,29 defined immunological failure based on previous WHO guidelines as a decrease of CD4 + cell count compared to baseline or below the suppression of immunity (CD4 + count < 200 cells / µL) 28.

Among patients identified as failure, 66.26% were switched to first line of second intention and 6.5% were switched to second line of ART. This is faring much more than the record for the southern study of Ethiopia and Tanzania, where respectively 15% 13 and 41% 30 of failed patients initiated second-line ART. This might show that patients who fail the regimen are not switched in a timely manner to the correct regimen, in other words, there is a delay to moving from the failed line to the next line. Consequently, we found a significant frequency of patients in the advanced stage (763 patients in stage III / IV) and we also identified 64 AIDS-related deaths in the last stage and had opportunistic infections (tuberculosis, toxoplasmosis, cryptococcal meningitis, cancer). These deaths due to clinical, immunological and virological failures (In addition, people living with HIV present to treat an advanced illness, with a low CD_4_ count and a high risk of serious illness and death). An indication of this fact in this study is that among the study subjects who died 64 (5.88%) had clinical, immunologic and virologic insufficiency, only 6.7% of them had moved second line ART, and deceased patients were more likely to have immunologic and virologic failure than those who were alive and receiving treatment at the time of the study. Studies have shown that putting patients on a failing first-line regimen leads to high morbidity and mortality 31-33.

This study shows that the early initiation of a treatment regimen after the diagnosis of failure is key for the reduction of morbidity and mortality associated with taking a failing regimen. This result has been proven by the WHO (2010) 33. Several studies reported that starting ART late in patients with advanced immunosuppression is associated with antiretroviral treatment failure 18, 34-39.

Among the patients with immune-virological failure, a higher frequency of death was observed than that of the patients without immune-virological failure. WHO (2010) 34 and several other studies 40-42 recommended immunological success as a surrogate marker for the virological suppression. In the current study, 24% of patients had immunological failure (IF); while, 76% of patients had immunological success and 69.05% of patients had virological suppression. This (rather low) performance of virological suppression is less than the current goal of virological suppress of the UNAIDS 90-90-90 43 (diagnose 90% of HIV-infected persons worldwide; link 90% of these to antiretroviral therapy (ART); and achieve 90% virologic suppression among ART recipients 44, 45.

In this study, 8 (0.74%) patients with virological failure, had CD4> 500 Cells / mL and a viral load > 1000 copies / mL. Here predictive precision is low, immunological success overestimates virological suppression 46, 47. It is not always a real indicator to assess virological failure. Indeed, the replacement of immunological surveillance by virological surveillance in non-viremic patients in a gradual manner will minimize the costs associated with dual immunovirological surveillance. Therefore, the HIV plasma viral load test should be available to regularly monitor patients at least once every six months to diagnose virological failure early, before the development of drug-resistant mutations, and to initiate interventions. The use of GeneXpert for HIV viral load testing 48 sis also another option for resource-limited countries.

WHO urges action against HIV drug resistance threat. The WHO HIV drug resistance report 1 shows that in 6 of the 11 countries surveyed in Africa, Asia and Latin America, over 10% of people starting antiretroviral therapy had a strain of HIV that was resistant to some of the most widely used HIV medicines. Once the threshold of 10% has been reached, WHO recommends those countries urgently review their HIV treatment programs.

In treated patients, a frequency of resistance-associated mutations is estimated at approximately 80% in the United States and 50 to 80% in Europe; the most frequent for the NRTI are the M184V and the mutations of the thymidine analogs, M41L, D67N and T215Y / F. In the European cohort, the prevalence of resistance was estimated to be non-NNRTI, NRTI and protease inhibitors, 43%, 15% and 25%, respectively 39,49.

In Latin America, the studies are heterogeneous, which is why it is difficult to systematically analyse the frequency of resistance. In South America - Brazil, Argentina and Chile - it has been determined that the secondary resistance for some antiretroviral is between 80 and 90%; in Brazil, mainly in the NRTI group, and in Argentina and Chile, for NNRTIs and protease inhibitors. Among the most frequent mutations is M184V, followed by mutations of thymidine analogs; in non-nucleoside inhibitors, K103N, and for protease inhibitors, mutations at positions 63, 90 and 82 50.

To reach the 90-90-90 target, the HIV viral plasma load test must be available and accessible at least once every six months, regular follow-up with patient awareness and if it fails, we must look for the causes and also the availability of the resistance typing test.

## Conclusion

The extent of therapeutic, virological and immunological failure was high. To limit these problems, early detection of the virus (HIV), and initiation of ART as early as possible before the occurrence of a severe immune suppression, the management of these failures and the improvement of support mechanisms are recommended. In conclusion, the majority of patients diagnosed early respond immunologically and virologically to the firstline ART. Our study shows that immunological monitoring alone is insufficient to predict virological suppression and therapeutic success. Consequently, we recommend the accessibility of the test of HIV viral plasma load as a routine exam. This test is sensitive and precise enough to evaluate the therapeutic success, to detect the virological failure early and to prevention of drug-resistant.

## Data Availability

The data presented in this study are available on request from the corresponding author.
